# New Vitamin and Mineral Supplements: Acceptability, Tolerability, and Short-Term Adherence in a Paediatric Cohort with IMDs

**DOI:** 10.3390/nu18203309

**Published:** 2026-10-09

**Authors:** Martina Tosi, Sharon Evans, Ozlem Yilmaz Nas, Anne Daly, Alex Pinto, Julia Ackrill, Catherine Ashmore, Anita MacDonald

**Affiliations:** Department of Dietetics, Birmingham Women’s and Children’s Hospital, Birmingham B4 6NH, UK; sharon.morris6@nhs.net (S.E.); ozlem.yilmaz@nhs.net (O.Y.N.); a.daly3@nhs.net (A.D.); alex.pinto@nhs.net (A.P.); julia.ackrill@nhs.net (J.A.); catherine.ashmore@nhs.net (C.A.); anita.macdonald@nhs.net (A.M.)

**Keywords:** inherited metabolic disorders, restricted diet, vitamin supplementation, mineral supplementation, multivitamin, micronutrient deficiency, product acceptability, palatability, children

## Abstract

**Background/Objectives:** Dietary therapy for inherited metabolic disorders (IMDs) can restrict nutrient-dense foods and increase reliance on vitamin and mineral supplementation. However, formulation-related barriers may limit supplement acceptability and adherence, particularly in children with feeding difficulties and complex dietary regimens. We evaluated the acceptability, gastrointestinal (GI) tolerability, and short-term adherence of two novel low-carbohydrate, low-volume vitamin and mineral formulations in children and adolescents with IMDs. **Methods:** This prospective, single-centre, open-label, non-randomised interventional study enrolled children and adolescents with IMDs aged ≥1 year who were either taking vitamin and mineral supplements already or were clinically identified as requiring micronutrient supplementation. Participants replaced their usual vitamin and mineral supplement with one of two age-appropriate study formulations (product A or B) for 7 consecutive days under usual home conditions. Acceptability was assessed using structured questionnaires, GI symptoms were assessed at baseline and on day 7 and monitored using daily diaries, and adherence was recorded daily by caregivers. Clinical characteristics and anthropometric measurements were assessed at baseline. **Results:** Thirty-one participants (median age, 7.2 years; range, 2.1–16.6) were enrolled; 15 were allocated to study product A and 16 to study product B, with 14 and 15 participants, respectively, completing the 7-day intervention. Full-dose intake (always or mostly) during the 7-day study period was reported in 27/30 (90.0%) participants. Among evaluable participants, GI symptoms improved or remained unchanged in 12/13 (92.3%) receiving product A and in 12/14 (85.7%) receiving product B. Mean acceptability scores improved for both product A (from 2.4 to 1) and product B (from 2.2 to 0.4), with lower scores indicating greater acceptability. Overall preference for the study product was reported by 10/14 (71.4%) participants receiving product A and 15/15 (100%) receiving product B. At the end of the study, 6/14 (42.9%) participants receiving product A and 7/15 (46.7%) receiving product B elected to continue to use the study product. **Conclusions:** In this exploratory study, two low-carbohydrate, low-volume vitamin and mineral formulations demonstrated favourable short-term acceptability, GI tolerability, and adherence in children and adolescents with IMDs. These findings suggest that formulation characteristics and ease of administration may influence the practical feasibility of micronutrient supplementation in this population. However, the short seven-day study duration and absence of a control group limit interpretation of these findings. Larger, longer-term studies are warranted to determine whether these benefits are sustained and whether improved acceptability and adherence translate into improved micronutrient status and clinical outcomes.

## 1. Introduction

Dietary therapy is central to the management of many inherited metabolic disorders (IMDs), maintaining metabolic stability, supporting normal growth and development, and reducing the risk of acute decompensation and long-term complications [1]. Depending on the underlying metabolic defect, treatment may require restriction or modification of protein or specific amino acids [2,3,4,5,6,7,8], total fat or selected lipid fractions [9,10,11], galactose [12] or carbohydrate intake [13,14]. Although essential, these highly restrictive diets may restrict nutrient-dense foods and reduce habitual dietary sources of vitamins and minerals [15,16,17,18,19]. Consequently, ensuring adequate micronutrient provision is an integral component of nutritional management in many IMDs.

This is particularly important during childhood, when dietary treatment may begin within the first days or weeks of life and coincides with periods of rapid growth, neurodevelopment and acquisition of feeding skills [20,21,22,23]. Young children have relatively high micronutrient requirements and limited physiological reserves, while acute illness and metabolic instability may further compromise oral intake [15,24,25,26,27]. Nutritional management is largely caregiver-dependent and becomes embedded within everyday feeding practices [28,29]. The transition from formula feeding to complementary foods represents an additional challenge, as dietary restrictions and continued reliance on specialised metabolic formulas may influence food exposure, sensory experiences and food acceptance during a critical developmental period [20,30,31].

Feeding difficulties may compound these nutritional challenges. Selective eating, reluctance to accept unfamiliar foods and persistent feeding difficulties have been described in children with IMDs [21,30,32]. Recurrent hospitalisation, gastrointestinal (GI) symptoms, enteral tube feeding and episodes of metabolic illness may further disrupt the development of oral feeding skills [30], while caregiver anxiety surrounding dietary management may unintentionally restrict opportunities for food exploration and normal feeding development [33]. As children become older, increasing their autonomy at school and in social environments, developmental changes and the progressive transfer of treatment responsibility from caregivers to the child introduce further barriers to maintaining complex dietary regimens [34,35]. Poor palatability, limited product choice, treatment monotony and the social visibility of specialised nutritional products may further increase treatment burden and compromise adherence [26,36,37,38,39]. Establishing nutritional regimens that are both clinically effective and acceptable is therefore important for sustaining treatment throughout childhood and into adolescence and adulthood.

Micronutrient provision within these complex dietary regimens is not straightforward. Many disease-specific metabolic formulas are fortified with vitamins and minerals [40], but their micronutrient composition varies and individual requirements may change with age, growth, clinical status, dietary prescription, pharmacological treatment or organ transplantation. Changes in metabolic formula intake, dietary liberalisation or treatment strategy may therefore substantially alter micronutrient exposure and increase dependence on separately prescribed vitamin and mineral supplements [15,41]. Importantly, suboptimal micronutrient intake or biochemical status has been reported across several IMDs despite specialist dietary management [33,42,43,44,45,46]. Given the roles of micronutrients in growth, immune function, bone health, erythropoiesis, energy metabolism and neurocognitive development [47,48,49,50], maintaining adequate provision throughout childhood and adolescence is an important component of long-term care [51].

However, the clinical effectiveness of micronutrient supplementation depends not only on nutrient composition but also on whether the prescribed preparation can be consistently administered and consumed. Existing vitamin and mineral formulations may present practical challenges for children with IMDs. Some contain appreciable quantities of carbohydrate, limiting their suitability for disorders requiring carbohydrate restriction or careful carbohydrate control, while scoop-based preparation may introduce dosing variability and increase caregiver burden [52,53]. Age restrictions, narrow flavour choice, poor palatability, undesirable texture, large administration volumes, poor solubility and limited compatibility with enteral feeding systems may further compromise usability and acceptability [16,18,54,55]. These constraints may be especially important for children who already have selective eating or feeding difficulties and for families managing multiple components of a demanding daily treatment regimen. Supplementary Table S1 summarises the nutritional composition of vitamin and mineral supplements currently available on prescription through the Advisory Committee on Borderline Substances (ACBS) in the United Kingdom (UK).

Despite the clinical importance of micronutrient supplementation, evidence evaluating stand-alone vitamin and mineral preparations in children and adolescents with IMDs is scarce. Previous research has identified difficulties in achieving prescribed micronutrient supplement intake in this population [55], suggesting that appropriate nutrient composition alone does not ensure successful treatment. Yet little is known about the extent to which formulation characteristics, including palatability, administration volume, preparation requirements and compatibility with disease-specific dietary restrictions, determine acceptability and adherence. This represents an important evidence gap because even nutritionally appropriate supplementation is unlikely to achieve its intended clinical benefit if it cannot be reliably incorporated into everyday treatment.

Therefore, this study aimed to evaluate the acceptability, gastrointestinal tolerability, and short-term adherence of two low-carbohydrate, low-volume vitamin and mineral formulations specifically designed for children and adolescents with IMDs.

## 2. Materials and Methods

### 2.1. Study Design

This prospective, single-centre, open-label, non-randomised interventional study evaluated two novel low-carbohydrate vitamin and mineral supplements in children with IMDs attending a specialist metabolic centre in the UK. Eligible participants, according to the age indication of the assigned study product, used the allocated product for 7 days under usual home conditions. The primary outcomes were product acceptability and GI tolerance. The study was registered at ClinicalTrials.gov (NCT07619170).

### 2.2. Inclusion and Exclusion Criteria

Children and adolescents with a confirmed diagnosis of an IMD were eligible if they met the age requirement for at least one of the study products (aged ≥1 year for study product A and ≥3 years for study product B) and were either receiving vitamin and mineral supplementation, orally or via an enteral feeding tube, or had been clinically identified as requiring micronutrient supplementation. Participants were also required to be willing and able to complete the 7-day study procedures. Exclusion criteria included age below the minimum requirement for the respective product; severe acute illness that could interfere with study participation or outcome assessment; poor adherence to prescribed dietary management; participation in another clinical trial within the preceding 2 weeks; antibiotic use within 2 weeks before enrolment; or concerns from the lead dietitian regarding the participant’s ability to adhere to the study protocol.

### 2.3. Study Products

Two low-carbohydrate vitamin and mineral formulations were evaluated according to age: UNO EasiVits (Easi Nutrition, London, UK; study product A) was suitable for children aged ≥1 year, while DUO EasiVits (Easi Nutrition, London, UK; study product B) was suitable for children aged ≥3 years. As the study was non-randomised, participants who were eligible for both formulations were allocated to study product A or B by the metabolic dietitian based on their clinical knowledge of the individual participant, including their dietary patterns, nutritional needs, particularly micronutrient requirements, growth and clinical characteristics. Both products were unflavoured, unsweetened, low-volume powdered formulations containing vitamins, minerals and trace elements, developed for individuals requiring therapeutic dietary restriction or additional micronutrient supplementation. Study product A was supplied in single-use 3 g sachets and formulated with reference to the Recommended Nutrient Intakes (RNIs) for children aged 1–3 years [56], with dosing for older children guided by age-appropriate micronutrient requirements. Each sachet was fully soluble in a minimum of 30 mL water and could be consumed immediately after preparation or mixed with a permitted cordial or other suitable beverage. Study product B was supplied in single-use 5 g sachets and formulated with reference to age-specific RNIs for children aged 3–11 years [56]. Each 5 g sachet was fully soluble in a minimum of 60 mL water and could be consumed using the same administration methods described for the 3 g sachet. The composition and key specifications of both formulations are presented in Table 1, with micronutrient provision relative to age-specific RNIs provided in Supplementary Table S2.

### 2.4. Study Procedures

At baseline (T0), participants were assessed by a specialist IMD dietitian, who introduced the assigned study formulation and provided individualised instructions for its preparation and administration. Participants either replaced their usual vitamin and mineral supplement with the assigned formulation for 7 days or, if they were not taking a supplement at baseline, started the assigned formulation for 7 days, administered once daily as either one 3 g sachet (study product A) or one 5 g sachet (study product B), with water or another permitted beverage. All other aspects of prescribed dietary management remained unchanged. Products were administered under usual home conditions to reflect routine use. Participants continued their usual therapeutic dietary management, specialised nutritional products and prescribed medications throughout the 7-day study period. The only change to participants’ usual regimen was the replacement of their usual vitamin and mineral supplement with the assigned study product, where applicable. Participants who were not receiving supplementation at baseline initiated the assigned study product according to their individual clinical requirements.

### 2.5. Assessments

Participant demographic and clinical characteristics were recorded at baseline (T0).

#### 2.5.1. Anthropometry

Body weight and height were measured at T0 by trained dietitians using standardised procedures. Height was measured to the nearest 0.1 cm using a Seca stadiometer, and body weight to the nearest 0.1 kg using calibrated digital scales (Model 875; Seca Medical Measuring Systems and Scales, Birmingham, UK).

#### 2.5.2. Dietary and Nutritional Assessment

Baseline dietary assessment included prescribed dietary management, feeding behaviour, use of specialised nutritional products, and current micronutrient supplementation. Information on prescribed vitamin and mineral supplements was obtained from clinical records and included product, formulation, prescribed and reported intake, and clinical indication.

Micronutrient supplementation was classified according to its intended clinical purpose: (1) comprehensive supplementation in children aged 1–3 years requiring a complete vitamin and mineral supplement; (2) comprehensive supplementation in children aged >3 years with low body weight or body size, for whom micronutrient requirements were considered comparable to those of younger children; or (3) supplementary (“top-up”) provision in children requiring additional vitamins and minerals to support achievement of age-appropriate RNIs. Classification as comprehensive or supplementary supplementation was based on the clinical indication documented by the prescribing dietitian.

Feeding behaviour and patterns of dietary restriction were documented following clinical assessment by the dietitian. Restrictive eating behaviour was defined as a limited dietary repertoire or reduced food acceptance, including avoidance of specific food groups or reliance on a restricted range of accepted foods. A documented clinical diagnosis of avoidant/restrictive food intake disorder (ARFID) was recorded separately. Participant demographics were recorded at study T0.

#### 2.5.3. Dietary Intake Assessment

At T0, dietary intake was assessed by the specialist IMD dietitian using a study-specific non-validated Rapid Dietary Intake Screening Tool, together with a 24 h dietary recall; the latter was used to corroborate the screening tool data. The screening tool comprised five domains: (A) high-protein foods, (B) starchy foods, (C) special low-protein foods (SLPFs), (D) protein substitutes, and (E) vitamin and mineral supplements. The tool provided a structured assessment of habitual protein-containing food and specialised product use and was specifically designed to identify reduced or self-restricted protein intake.

Usual intake of relevant foods and dietary products was recorded using predefined portion sizes and frequency-of-consumption categories, supported by standardised portion-size photographs. Reference portions were 30 g cooked meat or fish, 100 mL regular milk, one egg, 120 g yoghurt or dairy dessert, 30 g regular cheese, 60 g cooked pulses, and 30 g nuts or seeds. Sections relating to disease-specific dietary products, including protein substitutes and SLPFs, were completed only where applicable to the participant’s prescribed dietary management.

Participants were instructed to maintain their usual dietary practices throughout the 7-day intervention period, but to discontinue their usual vitamin and mineral supplement and replace it with the assigned study formulation for the duration of the intervention.

#### 2.5.4. Adherence Assessment

Adherence to the assigned study product was assessed using a structured daily diary completed by caregivers throughout the 7-day study period. Adherence was evaluated using two measures: frequency of consumption of the full prescribed dose and proportion of the prescribed daily dose consumed. Caregivers recorded the frequency with which the full dose was taken using six categories: never, very rarely, rarely, occasionally, mostly or always. The proportion of the prescribed daily dose consumed was recorded as 0%, 25%, 50%, 75% or 100%.

#### 2.5.5. Clinical Assessment and Gastrointestinal Tolerance

Clinical assessments were conducted at T0 and day 7 (T1). Medical history, general health status and concomitant medications were documented at T0. At T1, caregivers were systematically asked about any participant intercurrent illness, changes in health status, new or worsening symptoms, and any suspected adverse reactions associated with the study product.

GI symptoms were assessed at T0 and T1 using a structured symptom checklist completed by participants and/or caregivers, as age appropriate. The presence and severity of diarrhoea, constipation, bloating or abdominal distension, nausea, vomiting, burping or regurgitation, flatulence, and abdominal pain or discomfort were recorded. Each symptom was graded using a four-point ordinal scale (none, mild, moderate or severe). GI symptoms were additionally monitored throughout the 7-day study period using a daily caregiver-completed diary, enabling assessment of symptom occurrence and changes during product use.

#### 2.5.6. Acceptability

Product acceptability was assessed using a non-validated study-specific structured questionnaire completed by caregivers. As no validated tool was available to assess the acceptability of vitamin and mineral supplements in children with IMDs, this study-specific questionnaire was developed to evaluate sensory and practical aspects of the formulations relevant to their use in clinical practice. At (T0), participants receiving a vitamin and mineral supplement evaluated their usual supplement, and at T1, participants evaluated the assigned study product. Acceptability was summarised descriptively at each time point; because T0 and T1 data were not available for all participants, the number of participants contributing data differed between time points. Seven formulation-specific sensory and practical attributes were assessed: appearance, smell, taste, texture, ease of preparation, ease of consumption, and aftertaste. Each attribute was rated using a five-point ordinal scale ranging from ‘great’ (0) to ‘terrible’ (4), with lower scores indicating greater acceptability. A ‘not applicable’ response option was also provided; these responses were treated as missing and excluded from the analysis.

### 2.6. Ethical Approval

The study was approved by the London–Camberwell St Giles Research Ethics Committee (REC reference: 25/PR/1685; IRAS ID: 363225; 8 January 2026) and conducted in accordance with the Declaration of Helsinki. Written informed consent was obtained from parents or legal guardians before enrolment, with assent obtained from children where age- and developmentally appropriate. Participants and their caregivers were informed of their right to withdraw from the study at any time without consequence.

### 2.7. Statistical Analysis

No formal sample size calculation was performed. Given the exploratory nature of the study and the limited number of eligible participants available at the specialist metabolic centre, the sample size was determined by the number of participants enrolled during the recruitment period. Owing to the small sample size, analyses were descriptive and no inferential statistical testing was undertaken. Continuous variables are summarised using means or medians, as appropriate to their distribution. Ordinal variables are presented as medians with first and third quartiles (Q1–Q3). For the non-validated acceptability rating scales, median (Q1–Q3) scores are reported as the primary summary measure, with mean scores additionally provided to facilitate descriptive comparison between T0 and T1. Categorical variables are presented as frequencies and percentages.

For the Rapid Dietary Intake Screening Tool, reported weekly frequency categories were converted to estimated mean daily intake. The midpoint of each frequency category was multiplied by the corresponding standard portion size and divided by seven. Estimated intakes are reported as g/day, mL/day or portions/day, as appropriate. For the open-ended category (>14 servings/week), intake was conservatively assigned a value of 14 servings/week.

## 3. Results

### 3.1. Participants

Thirty-one children and adolescents with IMDs were enrolled, of whom 29 (93.5%) completed the 7-day study. Fifteen participants were allocated to study product A (≥1 year) and 16 to study product B (≥3 years). In the study product A group, 14 participants (93.3%) completed the study, while 15 participants (93.8%) completed the study in the study product B group. Two participants (S14 and S21) did not complete the study. S14 initiated the study product A but subsequently refused to continue and withdrew from the study. S21 withdrew before commencing the study product B due to family circumstances and therefore provided no evaluable study data. Two additional participants (S13 and S24) completed the study but had insufficient data for the GI and acceptability outcomes and were therefore excluded from these specific analyses. Consequently, 13 participants in the study product A group and 14 in the study product B group were included in the GI and acceptability analyses. Participant flow and outcome-specific analysis populations are presented in Figure 1.

Overall, 20 participants (64.5%) were male and 11 (35.5%) female, with a median age of 7.2 years (range 2.1–16.6 years). Participants were of Asian (15/31, 48.4%), White European (15/31, 48.4%) and Arabic (1/31, 3.2%) ethnicity.

The most frequently represented diagnostic groups were urea cycle disorders (10/31, 32.3%), fatty acid oxidation and transport disorders (7/31, 22.6%) and glycogen storage diseases (7/31, 22.6%). Other diagnoses included organic acidaemias (3/31, 9.7%), galactosaemia, maple syrup urine disease, homocystinuria and lysinuric protein intolerance (each 1/31, 3.2%). Three participants (9.7%) were newly diagnosed and nine (29.0%) had undergone liver transplantation.

Baseline characteristics are summarised in Table 2, with participant-level data provided in Supplementary Table S3.

### 3.2. Anthropometric Measurements and Growth Status

At baseline, growth concerns were identified in three participants on study product A (3/15, 20.0%). Two had markedly reduced linear growth and body weight, with height and weight z-scores of −3.30 and −2.50 in one participant and −5.26 and −5.30 in the other. A third participant had a height at the 5th centile and was undergoing clinical assessment for short stature (Table 2; Supplementary Table S4).

### 3.3. Dietary Assessment

Dietary management reflected the underlying IMD and individual nutritional requirements (Table 2). Dietary management data were available for 30 participants; one participant (S21) withdrew immediately after enrolment before dietary assessment. Nine participants (30.0%) followed a protein-restricted diet, five (16.7%) a carbohydrate-controlled diet and one (3.3%) a galactose-restricted diet, while 15 (50.0%) had no routine dietary restrictions.

A further three participants (10.0%) received modified uncooked cornstarch, contributing approximately 20%, 8% and 8% of estimated daily energy intake, respectively; one also received a glucose polymer supplement. One participant (3.3%) received medium-chain triglyceride supplementation three times daily. Four (13.3%) used special low-protein milk replacements. Two participants (6.7%) received supplementary enteral tube feeding, and one (3.3%) was exclusively enterally fed. No participants received protein substitutes.

Feeding difficulties and restricted food acceptance were highly prevalent. Eight participants (26.7%) had a documented diagnosis of ARFID, and a further 21 (70.0%) exhibited restrictive eating behaviours or limited food acceptance without a documented ARFID diagnosis, based on clinical dietetic assessment (Table 2). One participant (3.3%) received a tube-fed blended diet.

Habitual intake of protein-containing foods, assessed using the Rapid Intake Screening Tool, varied across the cohort (Table 2). Dietary intake data were available for 29 participants (S09 not recorded). Twenty-six (89.7%) consumed an estimated ≤45 g/day of cooked meat and fish, and 20 (69.0%) consumed ≤100 mL/day of cow’s milk. Intake of eggs, yoghurt, cheese, pulses, nuts and seeds was also limited, with some participants reporting no habitual consumption of these foods. Twenty-three of 29 participants (79.3%) consumed an estimated 0.5–1.5 portions/day of fruit and vegetables.

### 3.4. Micronutrient Supplementation and Study Product Adherence

At baseline, 9 of 30 participants (30.0%) were not receiving vitamin and mineral supplementation. Reasons included recent IMD diagnosis (*n* = 3), recent liver transplantation (*n* = 2), previous refusal of supplementation (*n* = 1), and use of a blended enteral feeding diet (*n* = 1) (Table 3). Among the 21 participants (21/30, 70.0%) receiving supplementation, formulations comprised powders (12/21, 57.1%), chewable gummies (8/21, 38.1%), and oral liquids (1/21, 4.8.3%). Powdered supplements were administered as a drink, by oral syringe or via an enteral feeding tube. Eight participants (8/21, 38.1%) used over-the-counter preparations rather than prescribed products.

Reported use of baseline supplementation was variable. Of the 21 participants receiving a vitamin and mineral supplement, 13 (61.9%) reported consuming 100% of the prescribed amount when taken, although the reported frequency of consumption ranged from always to occasionally. Two (9.5%) consumed approximately 75% of the prescribed amount, six (28.6%) consumed ≤25%, including three who reported taking approximately 25% and three who reported taking none.

All 30 participants were prescribed one sachet of the assigned study product, once daily for 7 consecutive days (Table 4). The study product was administered as a comprehensive source of micronutrients in 11 participants (36.7%) and as supplementary (“top-up”) provision in 19 participants (63.3%), according to individual clinical requirements. Comprehensive provision was defined as use of the study product as the primary source of vitamin and mineral supplementation, whereas top-up provision referred to its use alongside other sources of micronutrient intake, including natural food and medical formula. Fifteen participants received study product A (3 g/sachet) and 15 received study product B (5 g/sachet).

The study product was most commonly administered in the morning (19/30, 63.3%). Twenty-two participants (73.3%) consumed the product with a drink, while two (6.7%) received it via an enteral feeding tube. Overall, 27/30 participants (90.0%) reported consuming the full prescribed dose always or mostly during the 7-day study period. Three (10.0%) reported taking the full prescribed dose rarely or never, including one participant who withdrew following initial refusal of the product.

### 3.5. Clinical Assessment

No intercurrent illnesses or adverse events considered related to the study products were reported during the 7-day study period. Concomitant medications remained unchanged from baseline (Supplementary Table S5).

### 3.6. Gastrointestinal Tolerance

GI tolerability was assessed in 27 participants (Figure 1). Three participants (11.1%) were receiving laxative treatment for constipation at baseline (Supplementary Table S5).

Among participants receiving study product A (*n* = 13), GI symptom severity was generally low at both time points (Table 5). At baseline (T0), 4/13 participants (30.8%) reported at least one moderate or severe GI symptom, including one participant (7.7%) who reported severe flatulence. At T1, 3/13 participants (23.1%) each reported one moderate GI symptom, with no severe symptoms reported. When scores across all GI symptoms were summed for each participant, the total GI symptom score decreased from T0 to T1 in 6/13 participants (46.2%), remained unchanged in 6/13 (46.2%), and increased in 1/13 (7.7%).

Among participants receiving study product B (*n* = 14), GI symptom severity was generally low at both time points (Table 5). At baseline (T0), 4/14 participants (28.6%) reported at least one moderate or severe GI symptom, including one participant (7.1%) who reported severe flatulence. At T1, the same four participants continued to report at least one moderate or severe GI symptom, including one participant (7.1%) with severe flatulence. When scores across all GI symptoms were summed for each participant, the total GI symptom score decreased from T0 to T1 in 5/14 participants (35.7%), remained unchanged in 7/14 (50.0%), and increased in 2/14 (14.3%). In both participants with an increase, the total GI symptom score increased by only one point.

### 3.7. Acceptability

Acceptability was assessed across seven sensory and practical attributes using a five-point ordinal scale, with lower scores representing greater acceptability. Acceptability data at T1 were available for 27 participants, of whom 18 (66.7%) were receiving a vitamin and mineral supplement at T0 and were therefore eligible for comparison of their usual supplement with the assigned study product; nine (33.3%) were not receiving vitamin and mineral supplementation at baseline (Figure 1).

For product A, acceptability ratings were available for eight participants for their usual supplement at T0 and 13 participants for the study product at T1 (Table 6). Across all seven domains, mean scores were lower at T1 than at T0 in the available data, suggesting greater acceptability of the study product. However, these results should be interpreted cautiously, as baseline data were not available for all participants and the number of participants contributing data differed between time points. The largest descriptive differences were observed for ease of taking (2.7 vs. 0.9), aftertaste (2.4 vs. 0.9), taste (2.3 vs. 1.0), and ease of mixing (2.2 vs. 0.8).

For product B, acceptability data were available from 10 participants for their usual supplement at T0 and from 14 participants following introduction of the study product at T1 (Table 6). Lower mean scores were observed at T1 across each of the seven domains compared with T0, with the available responses generally favouring the study product. As with product A, baseline and T1 assessments were not available for the same number of participants; therefore, these findings are considered descriptive rather than a direct comparison between matched participants.

At study completion, among the 20 participants who had been receiving vitamin and mineral supplementation before enrolment and completed the study, preference for the study product over the previous supplement was reported by 6/9 (66.7%) participants assigned to product A and 11/11 (100%) participants assigned to product B. When considering all participants with available preference data, irrespective of previous supplement use, 10/14 (71.4%) participants assigned to product A and 15/15 (100%) participants assigned to product B expressed a preference for the study product.

Following the 7-day study period, 6/14 participants (42.9%) who completed the study with product A chose to continue using it in place of their previous or planned micronutrient supplementation, while 7/15 (46.7%) who completed the study with product B chose to continue using it. Overall, 13/29 participants (44.8%) who completed the study chose to continue using one of the study products after the 7-day intervention.

## 4. Discussion

In this prospective, non-randomised interventional study, two novel low-carbohydrate, low-volume vitamin and mineral formulations demonstrated favourable short-term acceptability and GI tolerability in children and adolescents with IMDs requiring specialised nutritional management. Most participants consumed the prescribed dose on all or most study days, sensory and practical acceptability ratings were favourable, and GI symptoms generally remained stable or decreased during the 7-day study period. No adverse events considered related to either study product were reported. Notably, these findings were observed across a clinically heterogeneous cohort encompassing different IMDs, dietary prescriptions, feeding practices and micronutrient requirements, including a high prevalence of restrictive eating behaviours. Almost half of study completers (13/29, 44.8%) chose to continue one of the study products following the 7-day evaluation. Collectively, these findings support the short-term feasibility of stand-alone micronutrient supplementation in this population, although conclusions regarding sustained acceptability, adherence or nutritional efficacy cannot be drawn.

The study also identified considerable variability in micronutrient supplementation before enrolment. Almost one-third of participants were not receiving a vitamin and mineral supplement at baseline, while among those prescribed supplementation, reported intake was inconsistent and over one-third used over-the-counter preparations. By comparison, most participants consumed the full prescribed amount of the study product on all or most study days, and ratings across taste, odour, texture, aftertaste, ease of preparation and ease of consumption were favourable. These observations should not be interpreted as evidence that the study products improved adherence, given the non-comparative design, brief follow-up and potential influence of study participation and product novelty. They nevertheless illustrate an important distinction between the prescription of micronutrients and their practical consumption: nutritional adequacy depends not only on what is prescribed, but also on whether supplementation can be incorporated consistently into everyday routines [57,58,59].

This distinction is particularly relevant in IMDs, where nutritional treatment may be complex, restrictive and lifelong. Research in metabolic nutrition has predominantly focused on specialised formulas, protein substitutes, special low-protein foods and disease-specific dietary interventions [60,61,62], with comparatively less attention given to stand-alone micronutrient preparations and the practical characteristics that may influence their use [15,41]. Micronutrient supplementation may represent only one component of a substantial daily treatment burden encompassing dietary restriction, specialised nutritional products, biochemical monitoring and pharmacological therapy [63,64]. Consequently, formulation characteristics such as palatability, administration volume, preparation requirements and dosing frequency may be clinically relevant alongside micronutrient composition [26]. A nutritionally appropriate formulation has limited value if it cannot be consumed reliably.

Acceptability may be especially important for children with established feeding difficulties. Restrictive eating behaviours or limited food acceptance were highly prevalent in this cohort, with a subset having a documented diagnosis of ARFID [65]. Feeding difficulties in children requiring therapeutic diets are likely to be multifactorial and may reflect prolonged dietary restriction, sensory preferences, previous enteral feeding, neurodevelopmental factors and repeated medical interventions [18,30,66,67]. Introducing an additional nutritional product with an unacceptable taste, texture, volume or administration burden could further complicate management. The favourable short-term acceptance observed in the present study is therefore encouraging, particularly given the feeding complexity of the cohort. However, feeding behaviour was not a primary outcome and was not evaluated using a validated instrument; consequently, no association between restrictive eating behaviour and product acceptability or adherence can be inferred.

The heterogeneity of dietary management within the cohort also illustrates the changing nutritional needs of individuals with IMDs. Participants followed protein-restricted, carbohydrate-controlled, galactose-restricted and unrestricted diets, while some dietary prescriptions were adjusted following liver transplantation. Contemporary metabolic management is increasingly dynamic, with pharmacological therapies, organ transplantation, dietary liberalisation and changes in metabolic control modifying the degree and nature of dietary restriction over time [47,63,68,69,70]. Importantly, reduced dietary restriction does not necessarily ensure micronutrient adequacy, particularly where food variety remains limited or established feeding behaviours persist. As reliance on fortified metabolic products decreases, some individuals may require separate micronutrient supplementation to maintain appropriate intake [64]. Stand-alone formulations may therefore provide a flexible means of adapting micronutrient provision as dietary treatment evolves, rather than assuming that micronutrient requirements can be inferred solely from the degree of prescribed dietary restriction [15].

The low-carbohydrate composition of the study formulations may provide additional flexibility for disorders requiring carbohydrate restriction or careful carbohydrate management [71,72]. Minimising the carbohydrate contribution of a micronutrient supplement may enable supplementation without materially adding to prescribed carbohydrate intake. Low-volume, once-daily administration may similarly reduce practical treatment burden and could be advantageous as children become increasingly responsible for their own treatment during adolescence [26,34]. These potential benefits were not directly evaluated in the present study, however, and no conclusions regarding metabolic control or superiority over alternative formulations can be made regarding these potential benefits.

GI tolerability was generally favourable over the 7-day evaluation. Most participants experienced stable or lower GI symptom burden, with no nausea or vomiting reported during product use and no adverse events considered related to the study formulations. This is relevant because GI symptoms may adversely affect the acceptance and continued use of oral nutritional products [73,74]. Nevertheless, pre-existing GI symptoms were present in some participants, the observation period was brief, and symptom assessment relied on participant and caregiver reporting. Changes in GI symptoms should therefore be interpreted descriptively and do not provide evidence that either formulation improves GI function.

A strength of this study was its prospective evaluation under usual home conditions, thereby assessing product use within the context in which supplementation would ordinarily be administered. The inclusion of children with diverse IMDs, dietary prescriptions, feeding methods and existing supplementation practices reflects the heterogeneity encountered in specialist metabolic practice. The study also evaluated outcomes directly relevant to patients and caregivers but infrequently examined in other micronutrient studies, including sensory characteristics, ease of preparation and administration, adherence, preference and GI tolerability. Inclusion of participants with restrictive eating behaviours further enhances the clinical relevance of the findings.

Several limitations should be acknowledged. The small, single-centre sample limits generalisability and precluded inferential, subgroup and disorder-specific analyses. No formal sample size calculation was performed, reflecting the exploratory nature of the study and the limited number of eligible participants available at the specialist metabolic centre. Consequently, the study may have been underpowered to detect differences in GI tolerance or other outcomes. The non-randomised, non-comparative design precludes causal inference, and selection bias cannot be excluded, as families willing to participate may have been more motivated to trial a new supplement. Furthermore, the exclusion of participants with poor dietary adherence or concerns regarding their ability to follow the study protocol may have resulted in a study population more likely to adhere to the intervention, potentially limiting the generalisability of the adherence findings to the wider patient population.

Comparison with previous supplementation was complicated by heterogeneity in products, formulations, administration methods and clinical indications, and not all participants were receiving supplementation at baseline. Study participation, increased monitoring and product novelty may also have influenced short-term adherence and acceptability. Acceptability was assessed using a study-specific questionnaire that had not undergone formal validation, while adherence was predominantly participant- or caregiver-reported rather than objectively verified, introducing potential recall, response and social-desirability bias. Route of administration may also have influenced assessments of product acceptability, as sensory attributes such as taste, texture and aftertaste are less relevant when a product is administered enterally, but only two participants received the study product via an enteral feeding tube. However, the small number of enterally fed participants prevented exploration of whether GI tolerance differed according to administration route. Future longer-term studies should consider incorporating objective measures of adherence, such as product counts or weighing of unused product, alongside caregiver-reported measures.

The study was conducted within the context of usual therapeutic management, and no formal wash-out period was implemented. Concomitant medications, specialised nutritional products and dietary management were not standardised during the 7-day study period. Consequently, any changes in GI symptoms cannot be attributed solely to the study products, particularly in participants receiving concomitant treatments or nutritional products that may themselves influence GI tolerance.

The 7-day follow-up assessed initial rather than sustained use and cannot determine whether favourable acceptability or adherence would persist over time. Finally, biochemical markers of micronutrient status and detailed measures of dietary micronutrient intake were not evaluated; consequently, the study cannot establish nutritional efficacy, micronutrient adequacy or clinical benefit. Micronutrient bioavailability and absorption were not assessed; therefore, achieving an adequate micronutrient intake cannot be assumed to result in adequate absorption or nutritional status, particularly in participants with altered GI function or malabsorption [75].

Future studies should evaluate these formulations in larger, multicentre cohorts over longer periods and incorporate validated measures of acceptability, objective assessment of adherence and biochemical markers of micronutrient status. Longitudinal evaluation would help determine whether initial acceptability is sustained and whether simplified, low-volume supplementation translates into improved long-term micronutrient intake. Studies should also examine whether acceptability and adherence differ according to age, diagnosis, feeding behaviour, route of administration and degree of dietary restriction. Given the evolving therapeutic landscape of IMDs, understanding how micronutrient requirements change following dietary liberalisation, pharmacological treatment and transplantation will be increasingly important.

## 5. Conclusions

In conclusion, these two low-carbohydrate, low-volume vitamin and mineral formulations were feasible to administer and generally acceptable and well tolerated during 7 days of use in a heterogeneous cohort of children and adolescents with IMDs. The findings highlight the importance of considering not only micronutrient composition but also palatability, volume, preparation and ease of administration when selecting supplementation for children requiring complex nutritional management. Longer-term studies incorporating objective adherence and biochemical outcomes are required to determine whether these favourable short-term findings translate into sustained use and improved micronutrient adequacy.

## Figures and Tables

**Figure 1:**
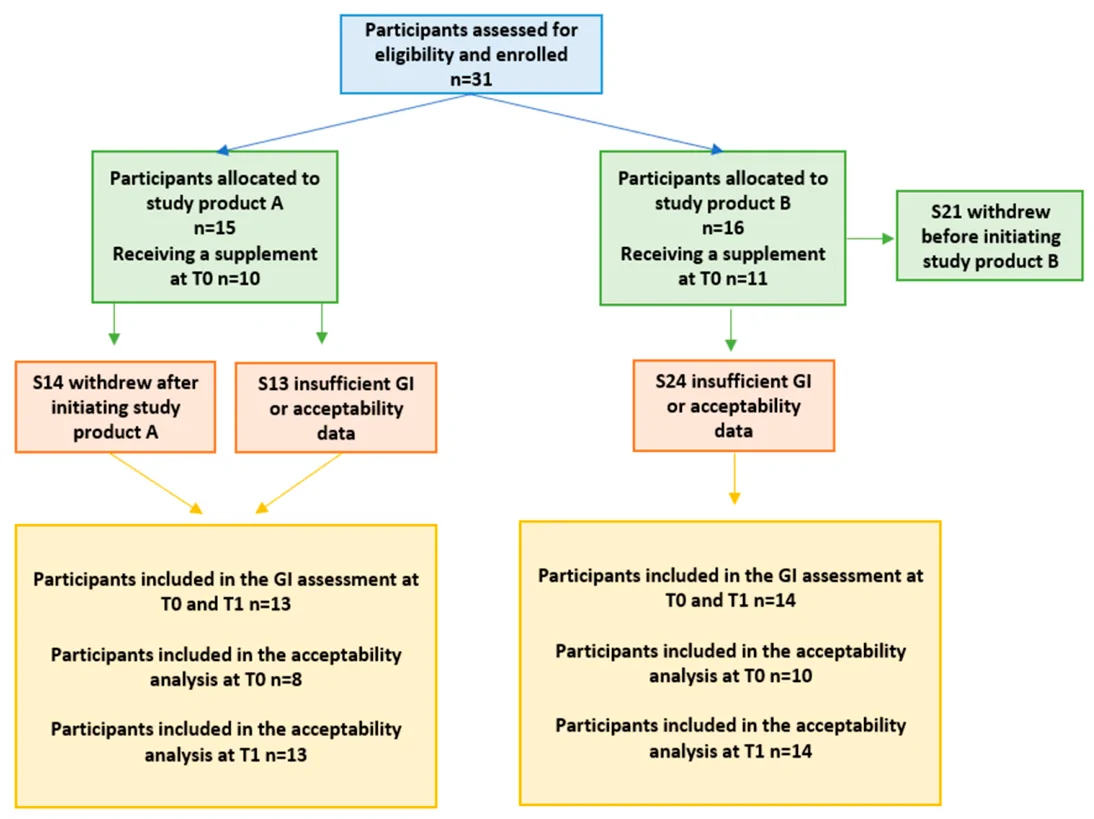
Participant flow and outcome-specific analysis populations. Abbreviations. S: subject; GI: gastrointestinal; T0: baseline; T1: day 7.

**Table 1:** Nutritional composition of the study products (*EasiVits UNO, EasiVits DUO*—*Easi Nutrition, London, UK*).

	EasiVits UNO		EasiVits DUO	
Component	Per 100 g	Per Sachet (3 g)	Per 100 g	Per Sachet (5 g)
Energy (kcal)	57.0	1.7	42.0	2.1
Energy (kJ)	238.0	7.0	176.0	8.8
**Macronutrients**				
Protein (g)	0.0	0.0	0.0	0.0
Carbohydrate (g)	3.46	0.1	5.4	0.27
of which sugars (g)	0.31	0.01	0.28	0.01
Fat (g)	0.07	0.0	0.05	0.0
Fibre (g)	21.4	0.64	10.0	0.5
**Vitamins**				
Vitamin A (µg)	15,000.0	450.0	10,000.0	500.0
Vitamin D (µg)	500.0	15.0	300.0	15.0
Vitamin E (mg α-TE)	200.0	6.0	180.0	9.0
Vitamin K (µg)	420.0	12.6	940.0	47.0
Vitamin C (mg)	1667.0	50.0	1000.0	50.0
Thiamin (mg)	17.0	0.5	20.0	1.0
Riboflavin (mg)	20.0	0.6	24.0	1.2
Niacin (mg NE)	267.0	8.0	260.0	13
Pantothenic Acid (mg)	100.0	3.0	80.0	4.0
Vitamin B6 (mg)	23.0	0.7	24.0	1.2
Folic Acid (µg)	3000.0	90.0	4000.0	200.0
Vitamin B12 (µg)	43.0	1.3	56.0	2.8
Biotin (µg)	500.0	15.0	2000.0	100.0
Choline	6000.0	180.0	5000.0	250.0
**Minerals**				
Sodium (mg)	0.41	0.01	0.62	0.03
Potassium (mg)	trace	trace	trace	trace
Chloride (mg)	trace	trace	trace	trace
Calcium (mg)	13,333.0	400.0	14,400.0	720.0
Phosphorus (mg)	9333.0	280.0	11,100.0	555.0
Magnesium (mg)	2833.0	85.0	4300.0	215.0
Iron (mg)	233.0	7.0	212.0	10.6
Zinc (mg)	167.0	5.0	160.0	8.0
Copper (mg)	18.0	0.55	18.0	0.9
Manganese (mg)	7.0	0.2	20.0	1.0
Molybdenum (µg)	333.0	10.0	740.0	37.0
Selenium (µg)	667.0	20.0	1000.0	50.0
Chromium (µg)	333.0	10.0	360.0	18.0
Iodine (µg)	2333.0	70.0	2200.0	110.0

Product ingredients EasiVits UNO: calcium phosphate, inulin, magnesium phosphate, choline bitartrate, calcium carbonate, vitamin C (ascorbic acid), ferric pyrophosphate, zinc sulphate, vitamin E acetate, vitamin B3 (nicotinamide), maltodextrin, vitamin B5 (calcium d-pantothenate), vitamin B6 (pyridoxine hydrochloride), vitamin B1 (thiamine hydrochloride), vitamin B2 (riboflavin), copper sulphate, manganese sulphate, vitamin A (retinyl acetate), folic acid, potassium iodide, sodium selenite, vitamin D3 (cholecalciferol), D-biotin, vitamin K1 (phylloquinone), chromium chloride, sodium molybdate, vitamin B12 (cyanocobalamin). Product ingredients EasiVits DUO: magnesium phosphate, calcium phosphate, choline bitartrate, inulin, calcium carbonate, vitamin C (ascorbic acid), ferric pyrophosphate, zinc sulphate, vitamin E acetate, vitamin B3 (nicotinamide), maltodextrin, vitamin B5 (calcium d-pantothenate), vitamin B6 (pyridoxine hydrochloride), vitamin B1 (thiamine hydrochloride), vitamin B2 (riboflavin), manganese sulphate, copper sulphate, vitamin A (retinyl acetate), folic acid, potassium iodide, sodium selenite, D-biotin, vitamin K1 (phylloquinone), sodium molybdate, vitamin D3 (cholecalciferol), chromium chloride, vitamin B12 (cyanocobalamin).

**Table 2:** Baseline dietary management, feeding behaviour, and nutritional characteristics of participants.

S	Age (Years)	Condition	Dietary Management and Growth Characteristics	Eating Behaviour and Food Acceptance Patterns	High-Protein Foods							Fruit and Vegetables (Portions/Day)
					Cooked Meat/Fish (g/Day)	Milk (ml/Day)	Egg (n/Week)	Yoghurt (g/Day)	Cheese (g/Day)	Cooked Pulses (g/Day)	Nuts (g/Day)	
S01	6.0	ASLD	Protein-restricted 1 g/kg/day SPLFs: milk daily Growth impairment ^1^	ARFID (10 foods accepted); minimal HBV protein foods	10	0	0	35	9	45	0	1.5
S02	2.1	Citrullinemia	Protein-restricted 1 g/kg/day	ARFID; minimal HBV protein foods	10	30	1	35	0	15	0	1.5
S03	5.4	ASLD	Protein-restricted 1 g/kg/day Severe growth impairment ^2^	Restrictive eater; minimal HBV protein foods	10	0	0	35	0	0	0	0.5
S04	10.5	GA 1	Normal diet	Restrictive eater	25	30	2.5	35	25	15	10	0.5
S05	14.7	SBCAD deficiency	Normal diet	Restrictive eater	25	30	>5	95	25	15	25	0.5
S06	3.2	Citrin deficiency	Carbohydrate-restricted MCT-supplemented	Restrictive eater	180	100	>5	35	60	15	25	2.5
S07	4.6	GSD Type VI	Carbohydrate-restricted with modified uncooked cornstarch and glucose polymer supplementation (~20% daily energy intake) Growth impairment ^3^	Restrictive eater	25	200	1	95	25	45	0	1.5
S08	7.3	MCADD	Normal diet	Restrictive eater	25	80	1	35	25	15	0	1.5
S09	2.9	Galactosaemia	Galactose-restricted	Restrictive eater	NR							
S10	2.9	IVA	Protein-restricted 1.5 g/kg/day SPLFs: milk daily	Restrictive eater	45	200	2.5	35	0	15	25	1.5
S11	2.8	MCT I deficiency (new diagnosis)	Normal diet	Restrictive eater	45	155	0	190	25	95	0	1.5
S12	4.5	MCT I deficiency (new diagnosis)	Normal diet	Restrictive eater	45	155	0	190	25	45	0	1.5
S13	4.2	MCADD	Normal diet	ARFID (5 foods accepted)	0	80	0	0	25	0	0	0.5
S14	3.1	GSD Type VI	Carbohydrate-restricted with modified uncooked cornstarch supplementation (~8% daily energy intake)	Restrictive eater	10	300	0	35	0	15	0	0.5
S15	3.8	OTC deficiency (new diagnosis)	Protein-restricted 1 g/kg/day	Restrictive eater; minimal HBV protein foods	0	80	2.5	35	0	15	0	1.5
S16	13.9	OTC deficiency post liver transplant	Normal diet Previously protein-restricted 0.9 g/kg/day	Restrictive eater; slow to resume intake during recovery	35	80	2.5	35	15	15	0	1.5
S17	8.4	MMA post liver transplant	Protein-restricted 40 g/day SPLFs: milk daily	ARFID (5–10 foods accepted); minimal HBV protein foods; fed by parents	10	0	2.5	35	0	45	0	0.5
S18	15.9	MSUD post liver transplant	Normal diet Previously protein-restricted 3 g/day	Restrictive eater (10 foods accepted)	95	30	5	0	10	0	0	0
S19	8.3	GSD Type I B post liver transplant	Normal diet but ongoing loose stools and slow to gain weight and height	Restrictive eater (10 foods accepted)	40	80	2.5	34	10	17	10	0.5
S20	12.5	GSD Type I B post liver transplant	Normal diet	Restrictive eater; minimal HBV protein foods	20	55	2.5	34	10	47	10	1.5
S21	12.8	HCU	withdrawn									
S22	7.2	OTC deficiency	Protein-restricted 1.5 g/kg/day; poor weight/height gain	ARFID (5–10 foods accepted); minimal HBV protein foods	0	0	7	0	10	47	0	0.5
S23	8.7	OTC deficiency post liver transplant	Normal diet Top-up tube feeding	ARFID (10 foods accepted); minimal HBV protein foods	10	500	1	35	0	0	0	1.5
S24	15.8	OTC deficiency post liver transplant	Normal diet, Top-up tube feeding, and chronic diarrhoea with poor weight gain	Restrictive eater	35	470	2.5	35	10	15	0	1.5
S25	13.5	Citrullinemia post liver transplant	Normal diet	ARFID (10–15 foods accepted); minimal HBV protein foods; fed by parents	15	55	2.5	35	10	45	0	1.5
S26	5.1	GSD Type IV post liver transplant	Normal diet	ARFID (5 foods accepted); minimal HBV protein foods	25	30	0	0	10	0	0	0
S27	8.8	GSD Type VI	Carbohydrate-restricted	Restrictive eater	60	0	5	190	10	15	10	1.5
S28	7.2	GSD Type VI	Carbohydrate-restricted with modified uncooked cornstarch supplementation (~8% daily energy intake)	Restrictive eater	45	80	2.5	95	25	15	0	2.5
S29	16.6	SBCAD deficiency	Protein-restricted 1 g/kg/day; blended diet; enterally tube fed	No oral food intake	45	200	2.5	95	0	15	10	1.5
S30	6.0	MCT I deficiency	Normal diet	Restrictive eater; food neophobia	0	155	0	190	45	0	0	0
S31	4.9	Lysinuric protein intolerance	Protein-restricted 1.3 g/kg/day SPLFs: milk daily	Restrictive eater	10	30	0	0	10	45	10	0

^1^ Height and weight z-scores: −3.3 and −2.5; ^2^ height and weight z-scores: −5.26 and −5.3; ^3^ short stature for age (height: 5th centile). Abbreviations: S: subject; ASLD: argininosuccinate lyase deficiency; GA1: glutaric aciduria type 1; SBCAD: short/branched-chain acyl-CoA dehydrogenase; GSD: glycogen storage disease; MCADD: medium-chain acyl-CoA dehydrogenase deficiency; IVA: isovaleric acidemia; MCT I: monocarboxylate transport 1; OTC: ornithine transcarbamylase; MMA: methylmalonic acidemia; MSUD: maple syrup urine disease; HCU: homocystinuria; ARFID: avoidant/restrictive food intake disorder; HBV: high biological value; MCT: medium-chain triglycerides; SLPFs: special low-protein foods; NR: not recorded (dietary data were unavailable for S09).

**Table 3:** Baseline multivitamin supplement prescription and adherence.

S	Baseline Supplement	Daily Dose	Administration Method	Full Dose Taken; Amount Taken
S01	Paediatric Seravit, Nutricia Ltd., Trowbridge, UK	15 g	Mixed with food	Very Rarely; 25%
S02	Fruitivits, Vitaflo, Liverpool, UK.	2.5 g (25 mL)	Orally via syringe	Never; 0%
S03	Fruitivits, Vitaflo UK.	1 × 6 g sachet	Mixed with fruit juice	Rarely; 25%
S04	Chewy Vites Kids Vitamin D3 (OTC)	1	Chewable gummies	Always; 100%
S05	Chewy Vites Kids Vitamin D3 (OTC) Previously refused Fruitivits	1	Chewable gummies	Always; 100%
S06	N/A	N/A	N/A	N/A
S07	Fruitivits, Vitaflo UK.	3 g (30 mL)	Orally via syringe	Occasionally; 100%
S08	Multivitamin Jellybeans (OTC)	1	Chewable gummies	Always; 100%
S09	N/A	N/A	N/A	N/A
S10	Paediatric Seravit, Nutricia Ltd.	15 g	Mixed with drink	Always; 100%
S11	N/A New diagnosis	N/A	N/A	N/A
S12	N/A New diagnosis	N/A	N/A	N/A
S13	Abidec Multivitamin Liquid Drops	0.6 mL	Orally via syringe	Mostly; 100%
S14	Boots Multivitamin Gummies (OTC)	1	Chewable gummies	Occasionally; 100%
S15	N/A New diagnosis	N/A	N/A	N/A
S16	N/A Recent liver transplant	N/A	N/A	N/A
S17	Fruitivits, Vitaflo UK.	1 × 6 g sachet	Orally via syringe	Always; 100%
S18	N/A Recent liver transplant	N/A	N/A	N/A
S19	Fruitivits, Vitaflo UK.	1 × 6 g sachet	Mixed with drink	Never; 0%
S20	Bassett Kids multivitamins (OTC)	1	Chewable gummies	Occasionally; 25%
S21	withdrawn			
S22	Paediatric Seravit, Nutricia Ltd.	15 g (1 × 5 g scoop TDS)	Mixed with drink	Mostly; 100%
S23	Boots Centrum Kids multi gummies (OTC)	1	Chewable gummies	Mostly; 100%
S24	Boots Centrum Kids multi gummies (OTC)	1	Chewable gummies	Never; 0%
S25	Boots Chewy Vites for Kids (OTC)	1	Chewable gummies	Mostly; 75%
S26	N/A Previous supplements refused	N/A	N/A	N/A
S27	Fruitivits, Vitaflo UK.	1 × 6 g sachet	Mixed with drink	Mostly; 100%
S28	Fruitivits, Vitaflo UK.	4 g (40 mL)	Orally via syringe	Mostly; 100%
S29	Fruitivits, Vitaflo UK.	1 × 6 g sachet	Via feeding tube	Always; 100%
S30	N/A Blended diet	N/A	N/A	N/A
S31	Phlexy-vits, Nutricia Ltd.	½ (3.5 g) sachet	Mixed with drink	Mostly; 75%

Abbreviations: S: subject; OTC: over the counter; N/A: not applicable; TDS: three times daily.

**Table 4:** Rationale for micronutrient supplementation, study product prescription and adherence.

S	Rationale for Micronutrient Supplement	Additional Considerations	Daily Dose Study Product	Administration Method	Full Dose Taken; Amount Taken
S01 ^1^	Comprehensive micronutrient provision	Weight corresponding to 3-year old child; low iron/calcium/ferritin/folate	1 sachet 3 g study product A	Drink and food	Always; 100%
S02	Comprehensive micronutrient provision	N/A	1 sachet 3 g study product A	No data	Mostly; 100%
S03 ^2^	Comprehensive micronutrient provision	Weight corresponding to 2-year old child; low iron/calcium/ferritin/folate	1 sachet 3 g study product A	Drink and food	Always; 100%
S04 *	Top-up micronutrient provision	N/A	1 sachet 3 g study product A	Drink	Always; 100%
S05 *	Top-up micronutrient provision	N/A	1 sachet 3 g study product A	Drink	Always; 100%
S06	Comprehensive micronutrient provision	N/A	1 sachet 3 g study product A	Drink and food	Very rarely; 25% £
S07 ^3^	Top-up micronutrient provision	Small for age	1 sachet 3 g study product A	Drink and food	Always; 100%
S08 *	Top-up micronutrient provision	N/A	1 sachet 3 g study product A	Drink	Always; 100%
S09	Top-up micronutrient provision	Diet naturally low in calcium and vitamin D	1 sachet 3 g study product A	Drink	Always; 100%
S10	Comprehensive micronutrient provision	N/A	1 sachet 3 g study product A	Drink	Always; 100%
S11	Top-up micronutrient provision	N/A	1 sachet 3 g study product A	Drink	Always; 100%
S12 *	Top-up micronutrient provision	N/A	1 sachet 3 g study product A	Drink	Always; 100%
S13 *	Top-up micronutrient provision	Severe dietary restriction requiring vitamin and mineral supplementation	1 sachet 3 g study product A	Drink	Never; 25% $
S14	Top-up micronutrient provision	N/A	1 sachet 3 g study product A	Drink	Never; 0% #
S15	Comprehensive micronutrient provision	Limited intake of iron/vitamin B12/folate/calcium	1 sachet 3 g study product A	Drink	Always; 100%
S16	Comprehensive micronutrient provision	Low micronutrient intake; previously low zinc/vitamin D ^	1 sachet 5 g study product B	Via feeding tube	Always; 100%
S17	Comprehensive micronutrient provision	Low micronutrient intake	1 sachet 5 g study product B	Drink	Always; 100%
S18	Top-up micronutrient provision	Limited micronutrient intake	1 sachet 5 g study product B	Drink	Always; 100%
S19	Top-up micronutrient provision	Low vitamin D/ferritin/folate/iron	1 sachet 5 g study product B	Drink	Always; 100%
S20	Top-up micronutrient provision	Low vitamin B12/ferritin/folate/vitamin D/calcium	1 sachet 5 g study product B	Drink	Always; 100%
S21	withdrawn				
S22	Comprehensive micronutrient provision	Low iron/vitamin B12/ferritin/folate/calcium	1 sachet 5 g study product B	Drink	Mostly; 100%
S23	Top-up micronutrient provision	Limited intake of calcium/iron/vitamin B12	1 sachet 5 g study product B	Drink	Always; 100%
S24	Top-up micronutrient provision	Low iron/vitamin B12/ferritin/folate/calcium	1 sachet 5 g study product B	Drink	Always; 100%
S25	Top-up micronutrient provision	Low iron/vitamin B12/ferritin/folate/calcium	1 sachet 5 g study product B	Drink	Always; 100%
S26	Top-up micronutrient provision	N/A	1 sachet 5 g study product B	Drink	Always; 100%
S27	Top-up micronutrient provision	N/A	1 sachet 5 g study product B	Drink	Always; 100%
S28	Top-up micronutrient provision	N/A	1 sachet 5 g study product B	Drink and food	Mostly; 100%
S29	Top-up micronutrient provision	N/A	1 sachet 5 g study product B	Via feeding tube	Always; 100%
S30	Comprehensive micronutrient provision	Low micronutrient intake and food neophobia	1 sachet 5 g study product B	Drink	Always; 100%
S31	Comprehensive micronutrient provision	Low micronutrient intake	1 sachet 5 g study product B	Drink	Always; 100%

^1^ Height and weight z-scores: −3.3 and −2.5; ^2^ height and weight z-scores: −5.26 and −5.3; ^3^ short stature for age (height: 5th centile). * Normal diet but requiring small nutritional top-up; £ S06 tried for 2 days but refused; $ S13 tried for 3 days but then refused; # S14 tried but refused and withdrew. ^ Post transplant was very unwell and did not eat well. Abbreviations: S: subject; N/A: not applicable.

**Table 5:** Gastrointestinal symptoms (median, Q1–Q3) reported at T0 and T1 in participants receiving study product A (*n* = 13) and study product B (*n* = 14).

GI Symptoms	Product A (*n* = 13)				Product B (*n* = 14)			
	T0 Median (Q1–Q3)	T1 Median (Q1–Q3)	Moderate/Severe at T0, *n* (%)	Moderate/Severe at T1, *n* (%)	T0 Median (Q1–Q3)	T1 Median (Q1–Q3)	Moderate/Severe at T0, *n* (%)	Moderate/Severe at T1, *n* (%)
Diarrhoea	0 (0–0)	0 (0–0)	0	0	0 (0–0)	0 (0–0)	1 (7.1%)	1 (7.1%)
Constipation	1 (0–1)	0 (0–1)	1 (7.7%)	2 (15.4%)	0 (0–0)	0 (0–0)	0	0
Bloating/abdominal distension	0 (0–1)	0 (0–0)	0	0	0 (0–1)	0 (0–1)	0	0
Nausea	0 (0–0)	0 (0–0)	0	0	0 (0–0)	0 (0–0)	0	0
Vomiting	0 (0–0)	0 (0–0)	0	0	0 (0–0)	0 (0–0)	0	0
Burping/regurgitation	0 (0–1)	0 (0–1)	0	0	1 (0–1)	1 (0–1)	1 (7.1%)	1 (7.1%)
Flatulence	1 (0–1)	0 (0–1)	2 (15.4%)	0	1 (0–1)	1 (0–1)	2 (14.3%)	2 (14.3%)
Abdominal discomfort/pain	0 (0–1)	0 (0–1)	1 (7.7%)	1 (7.7%)	0 (0–1)	0 (0–0)	1 (7.1%)	1 (7.1%)

Note. T0 = baseline; T1 = day 7; Q1 = first quartile; Q3 = third quartile.

**Table 6:** Acceptability ratings at baseline and day 7. Values are presented as median (Q1–Q3), with mean scores additionally reported for descriptive comparison. Characteristics were scored from Great (0) to Terrible (4).

Acceptability Domain	Product A (*n* = 13)		Product B (*n* = 14)	
	T0 (*n* = 8) Median (Q1–Q3) Mean	T1 (*n* = 13) Median (Q1–Q3) Mean	T0 (*n* = 10) Median (Q1–Q3) Mean	T1 (*n* = 14) Median (Q1–Q3) Mean
**Appearance**	1 (0–2) 0.9	0 (0–1) 0.8	2 (1–2.3) 1.9	0 (0–0) 0.2
**Smell**	2 (0–3.8) 1.9	1 (0–2) 1.1	2 (1.8–2.3) 2.0	0 (0–0) 0.1
**Taste**	3 (0–4) 2.3	1 (0.3–1) 1.0	2 (1.5–3) 2.2	0 (0–1) 0.4
**Texture**	2.5 (0.3–4) 2.3	1 (1–2) 1.4	2 (1.5–3) 2.2	0 (0–0) 0.1
**Ease of mixing**	2 (1.5–3) 2.2	1 (0.1) 0.8	2 (2–3) 2.3	0 (0–0) 0.1
**Ease of taking**	4 (0–4) 2.7	1 (0.1.5) 0.9	2 (1.8–2.3) 2.0	1 (0–1) 0.8
**Aftertaste**	4 (0–4) 2.4	1 (0–2) 0.9	2 (1.5–3) 2.1	1 (0–1) 0.9
**Overall**	3 (0.3–4) 2.4	1 (0–1.5) 1.0	2 (1.8–3) 2.2	0 (0–1) 0.4

Note. T0 = baseline; T1 = day 7; Q1 = first quartile; Q3 = third quartile. Descriptive statistics are based on all participants with available data at each time point; consequently, the number of participants contributing data differs between T0 and T1.

## Data Availability

The raw data supporting the conclusions of this article will be made available by the authors on request.

## Supplementary Materials

The following supporting information can be downloaded at: https://www.mdpi.com/article/10.3390/nu18203309/s1, Table S1: Nutritional details of micronutrients supplements available on prescription in the UK; Table S2: Comparison of UNO EasiVits and DUO EasiVits with UK Recommended Nutrient Intakes (RNI) for children and adolescents (Government Dietary Recommendations. Government recommendations for energy and nutrients for males and females aged 1–18 years); Table S3: Demographic information of participants; Table S4: Anthropometric data of participants at baseline; Table S5: Concomitant medications or nutritional supplements taken by participants.
