# Supplementary material for: New Vitamin and Mineral Supplements: Acceptability, Tolerability, and Short-Term Adherence in a Paediatric Cohort with IMDs
Source: Nutrients. 2026 Oct 9;18(20):3309. doi: 10.3390/nu18203309 (PMC13708595; doi:10.3390/nu18203309)
Supplement: Supplementary file 1 [file nutrients-18-03309-s001.zip › nutrients-4572908-supplementary.pdf]

Supplementary Table S1. Nutritional details of micronutrients supplements available on prescription in the UK.

Nutrients	Paediatric Seravit Powder		Fruitivits Sachets		Phlexy-Vits Sachets		Phlexy-Vits Tablets	
	Per 15g	Per 100g	Per 6g	Per 100g	Per 7g	Per 100g	Per 8.6g (5 tablets)	Per 100g
Energy kcal<br>Energy kJ	44<br>187	293<br>1246	2.4<br>10	40<br>168	1<br>4	15<br>63	5.1<br>20.4	59<br>237
Protein (g)	0	0	0	0	0.02	0.3	0	0
Carbohydrate (g)<br>Sugars (g)	11.3<br>0.88	75<br>5.9	0.5<br>0	8.3<br>0.5	0.04<br>0.04	0.5<br>0.5	0.05<br>0.03	0.6<br>0.4
Fat (g)	0	0	0	0	0	0	0.14	1.6
Fibre (g)	0	0	0.2	3.3	0.4	5.8	1.8	21
Vitamin A (µg)	480	3200	500	8333	800	11430	800	9295
Vitamin D (µg)	15	100	15	250	10	143	10	116
Vitamin E (mg α-TE)	8.1	54.2	9.3	155	9	129	9	104
Vitamin K (µg)	16.2	108	60	1000	70	1000	70	812
Vitamin C (mg)	60	400	40	667	50	715	50	580
Thiamin (mg)	0.48	3.2	1.2	20	1.2	17.1	1.2	14.1
Riboflavin (mg)	0.66	4.4	1.4	23	1.4	20	1.4	16.5
Niacin mg(mg NE)	6.2	41	15	250	20	286	20	232
Pantothenic Acid (mg)	-	24	4.7	78	5	71.5	5	58
Vitamin B6 (mg)	0.54	3.6	1.7	28	1.6	22.9	1.6	18.8
Folic Acid (µg)	90	600	240	4000	700	10000	700	8120
Vitamin B12 (µg)	1.3	9	2.8	47	5	71.5	5	58
Biotin (µg)	18	120	112	1867	150	2143	150	1739
Sodium (mg)	<6	<40	2.7	45	8.8	125	0	0
Potassium (mg)	<4.5	<30	11	180	<1.4	<20	0	0
Chloride (mg)	<4.5	<30	1.2	20	<0.35	<5	<3	<30
Calcium (mg)	413	2750	804	13400	775	14286	1000	11595
Phosphorus (mg)	278	1850	502	8367	775	11072	775	8985
Magnesium (mg)	75	500	201	3350	300	4286	300	3480
Iron (mg)	7.5	50	10	167	15.1	215	15.1	175
Zinc (mg)	5.4	36	10	167	11.1	158	11.1	129
Copper (mg)	0.63	4.2	1	17	1.5	21.5	1.5	17.5
Manganese (mg)	0.24	1.6	1.5	25	1.5	21.5	1.5	17.6
Molybdenum (µg)	16.2	108	68	1133	70	1000	70	812
Selenium (µg)	20.5	137	41	683	75	1072	75	870
Chromium (µg)	13.5	90	41	683	30	429	30	348
Iodine (µg)	123	820	169	2817	150	2143	150	1740

Supplementary Table S2. Comparison of UNO EasiVits and DUO EasiVits with UK Recommended Nutrient Intakes (RNI) for children and adolescents (Government Dietary Recommendations. Government recommendations for energy and nutrients for males and females aged 1 – 18 years).

Nutrient	unit	UNO EasiVits (per sachet, 3g)	DUO EasiVits (per sachet, 5g)	Recommended Nutrient Intake (RNI)						
				1-3 years	4-6 years	7-10 years	11-14 years Male	11-14 years Female	15-18 years Male	15-18 years Female
**Vitamin A**	ug	**450.00**	**500.00**	400.0	400.0	500.0	600.0	600.0	700.0	600.0
**Vitamin D3**	ug	**15.00**	**15.00**	10.00	10.0	10.0	10.0	10.0	10.0	10.0
**Vitamin E**	mg	**6.00**	**9.00**	ND	ND	ND	ND	ND	ND	ND
**Vitamin C**	mg	**50.00**	**50.00**	30.00	30.00	30.00	35.0	35.0	40.0	40.0
**Vitamin K***	ug	**12.60**	**47.00**	12.00	20.00	30.00	45.00	45.00	65.00	65.00
**Thiamin**	mg	**0.50**	**1.00**	0.30-0.40	0.7	0.7	0.9	0.7	1.1	0.8
**Riboflavin**	mg	**0.60**	**1.20**	0.60	0.8	1.0	1.2	1.1	1.30	1.10
**Niacin equivalents**	mg	**8.00**	**13.00**	4.70-7.20	11.0	12.0	15.0	12.0	18.0	14.0
**Vitamin B6**	mg	**0.70**	**1.20**	0.70	0.9	1.0	1.20	1.0	1.5	1.2
**Folic Acid**	ug	**90.00**	**200.00**	70.00	100	150	200	200	200	200
**Vitamin B12**	ug	**1.30**	**2.80**	0.50	0.8	1.00	1.2	1.2	1.5	1.5
**Biotin***	ug	**15.00**	**100.00**	20.00	25.00	25.00	35.00	35.00	35.00	35.00
**Pantothenic Acid***	mg	**3.00**	**4.00**	4.00	4.00	4.00	5.00	5.00	5.00	5.00
**Choline**	mg	**180.0**	**250.0**	140.0	170.0	250.0	340.0	340.0	400.0	400.0
**Sodium**	mg	**0.01**	**0.62**	800.00	1200	2000	2400	2400	2400	2400
**Potassium**	mg	**trace**	**trace**	800.00	1100	2000	3100	3100	3500	3500
**Chloride**	mg	**trace**	**trace**	800.00	1100	1800	2500	2500	2500	2500
**Calcium**	mg	**400.00**	**720.00**	350.00	450	550	1000	800	1000	800
**Phosphorus**	mg	**280.00**	**555.00**	270.00	350	450	775	625	775	625
**Magnesium**	mg	**85.00**	**215.00**	85.00	120.0	200.0	280.0	280.0	300.0	300.0
**Iron**	mg	**7.00**	**10.60**	6.90	6.1	8.7	11.3	14.8	11.3	14.8
**Copper**	mg	**0.55**	**0.90**	0.40	0.6	0.7	0.8	0.8	1.0	1.0
**Zinc**	mg	**5.00**	**8.00**	5.00	6.5	7.0	9.0	9.0	9.5	7.0
**Manganese**	mg	**0.20**	**1.00**	0.20	0.29	0.45	0.7	0.66	1.03	0.89
**Iodine**	ug	**70.00**	**110.00**	70.00	100.0	110.0	130.0	130.0	140.0	140.0
**Molybdenum***	ug	**10.00**	**37.00**	15.0	20.0	30.0	45.0	45.0	65.0	65.0

**Selenium**	ug	**20.00**	**50.00**	15.00	20.0	30.0	45.0	45.0	70.0	60.0
**Chromium**	ug	**10.00**	**18.00**	ND	ND	ND	ND	ND	ND	ND

*European Food Safety Authority (EFSA), Dietary Reference Values (DRV). ND: No dietary reference value established.

Supplementary Table S3. Demographic information of participants.

Subject	Age (yrs)	Diagnosis	Ethnic origin	Study product
S01	6	ASLD	White European	A
S02	2	Citrullinemia	Asian origin	A
S03	5	ASLD	White European	A
S04	11	GA 1	Asian origin	A
S05	15	SBCAD deficiency	Asian origin	A
S06	3	Citrin deficiency	Asian origin	A
S07	5	GSD Type VI	White European	A
S08	7	MCADD	White European	A
S09	3	Galactosaemia	White European	A
S10	3	IVA	Asian origin	A
S11	3	MCT I deficiency New diagnosis	White European	A
S12	5	MCT I deficiency New diagnosis	White European	A
S13	4	MCADD	White European	A
S14	3	GSD Type VI	Asian origin	A
S15	4	OTC deficiency New diagnosis	Asian origin	A
S16	14	OTC deficiency Post liver transplant	White European	B
S17	8	MMA Post liver transplant	Asian origin	B
S18	16	MSUD Post liver transplant	Asian origin	B
S19	8	GSD Type I B Post liver transplant	Asian origin	B

S20	13	GSD Type I B Post liver transplant	Arabic origin	B
S21	13	HCU	White European	B
S22	7	OTC deficiency	Asian origin	B
S23	9	OTC deficiency Post liver transplant	White European	B
S24	16	OTC deficiency post liver transplant	White European	B
S25	14	Citrullinemia Post liver transplant	Asian origin	B
S26	5	GSD Type IV Post liver transplant	White European	B
S27	9	GSD Type VI	Asian origin	B
S28	7	GSD Type VI	White European	B
S29	17	SBCAD deficiency	Asian origin	B
S30	6	MCT I deficiency	White European	B
S31	5	Lysinuric protein intolerance	Asian origin	B

Abbreviations: S. Subject; ASLD: argininosuccinate lyase deficiency; GA1: glutaric aciduria type 1; SBCAD: short/branched chain acyl-CoA dehydrogenase; GSD: glycogen storage disease; MCADD: medium chain acyl-CoA dehydrogenase deficiency; IVA: isovaleric acidemia; MCT I: monocarboxylate transport 1; OTC: Ornithine transcarbamylase; MMA: methylmalonic acidemia; MSUD: maple syrup urine disease; HCU: homocystinuria.

Notes. Participants' ages were rounded to the nearest whole number.

Supplementary Table S4. Anthropometric data of participants at baseline.

Subject	Age (yrs)	Weight at baseline (kg)	Height at baseline (cm)
S01[1]	6	15.6	100
S02	2	11.8	84.1
S03[2]	5	15.2	88
S04*	11	41.4	142.8
S05*	15	56.4	169
S06	3	14.2	90.6
S07[3]	5	17.0	97.2
S08*	7	28.0	120.7
S09	3	12.0	87.5
S10	3	15.2	94.5
11	3	15.8	91
S12*	5	20.2	105
S13*	4	16.5	105
S14	3	12.0	85
S15	4	17.5	105
S16	14	49.6	157
S17	8	26.6	117
S18	16	79.3	162
S19	8	21.2	115
S20	13	43.5	138.2
S21	13	97.4	156.9
S22	7	18.8	117.4
S23	9	29.2	133
S24	16	46.3	156
S25	14	53.9	154.3
S26	5	17.1	110
S27	9	24.8	115.1
S28	7	25.5	113
S29	17	37	146
S30	6	21.7	112.6
S31	5	16.8	104

Notes. [1]Height and Weight z-scores: -3.3 and -2.5 (size of a 3-year-old); [2]Height and Weight z-scores: -5.26 and -5.3 (on the 50th centile for a 2-year-old); [3]small for age, 5th centile for height; *normal diet but requiring small nutritional top-up.

Notes. Participants' ages were rounded to the nearest whole number.

Supplementary Table S5. Concomitant medications or nutritional supplements taken by participants.

Subject	Baseline	End of Study
S01	L-arginine supplementation; nitrogen-scavenging treatment	Unchanged
S02	L-arginine supplementation; nitrogen-scavenging treatment; laxative treatment	Unchanged
S03	L-arginine supplementation; nitrogen-scavenging treatment; vitamin D supplementation	Unchanged
S04	None	None
S05	None	None
S06	MCT-based nutritional supplement	Unchanged
S07	Modified uncooked corn-starch; glucose polymer	Unchanged
S08	None	None
S09	None	None
S10	Carnitine supplementation; glycine supplementation	Unchanged
S11	None	None
S12	Laxative treatment	Unchanged
S13	Laxative treatment; antiemetic treatment	Unchanged
S14	Modified uncooked corn-starch	Unchanged
S15	None	None
S16	Antiviral treatment; antiplatelet treatment; antihistamine treatment; citrulline supplementation; antibiotic treatment; antifungal treatment	Unchanged
S17	Vitamin D analogue treatment; antihypertensive treatment; magnesium supplementation; corticosteroid treatment; immunosuppressive treatment; carnitine supplementation; topical dermatological treatment	Unchanged
S18	Corticosteroid treatment; immunosuppressive treatment; antifungal treatment; antihypertensive treatment	Unchanged
S19	SGLT2 inhibitors; corticosteroid treatment; bile acid treatment; immunosuppressive treatment	Unchanged
S20	Immunosuppressive treatment; corticosteroid treatment; SGLT2 inhibitors	Unchanged
S21	Antihistamine treatment; methylation support treatment; folic acid supplementation; sleep-related treatment	Unchanged
S22	Citrulline supplementation; nitrogen-scavenging treatment; branched-chain amino acid supplementation	Unchanged
S23	Immunosuppressive treatment	Unchanged
S24	Immunosuppressive treatment	Unchanged

S25	Immunosuppressive treatment; hormonal treatment	Unchanged
S26	Immunosuppressive and corticosteroid treatment	Unchanged
S27	None	None
S28	Modified uncooked corn-starch	Unchanged
S29	Vitamin B12 supplementation; folic acid supplementation; proton pump inhibitor; bronchodilator; inhaled corticosteroid; muscle relaxant; antibiotic; antihistamine	Unchanged
S30	Iron supplementation	Unchanged
S31	Nitrogen-scavenging treatment	Unchanged
